# CD81 Is Essential for the Re-entry of Hematopoietic Stem Cells to Quiescence following Stress-Induced Proliferation Via Deactivation of the Akt Pathway

**DOI:** 10.1371/journal.pbio.1001148

**Published:** 2011-09-13

**Authors:** Kuanyin K. Lin, Lara Rossi, Nathan C. Boles, Brian E. Hall, Thaddeus C. George, Margaret A. Goodell

**Affiliations:** 1 Department of Pathology and Immunology, Baylor College of Medicine, Houston, Texas, United States of America; 2 Center for Cell and Gene Therapy, Baylor College of Medicine, Houston, Texas, United States of America; 3 Stem Cell and Regenerative Medicine Center, Baylor College of Medicine, Houston, Texas, United States of America; 4 Institute of Hematology and Medical Oncology “L. & A. Seràgnoli,” University of Bologna, S. Orsola-Malpighi Hospital, Bologna, Italy; 5 Amnis Corporation, Seattle, Washington, United States of America; 6 Department of Pediatrics, Baylor College of Medicine, Houston, Texas, United States of America; B.C. Cancer Agency, Canada

## Abstract

The regulatory mechanisms governing the cell cycle progression of hematopoietic stem cells (HSCs) are well characterized, but those responsible for the return of proliferating HSCs to a quiescent state remain largely unknown. Here, we present evidence that CD81, a tetraspanin molecule acutely responsive to proliferative stress, is essential for the maintenance of long-term repopulating HSCs. *Cd81* ^−*/*−^ HSCs showed a marked engraftment defect when transplanted into secondary recipient mice and a significantly delayed return to quiescence when stimulated to proliferate with 5-fluorouracil (5FU). In addition, we found that CD81 proteins form a polarized patch when HSCs are returning to quiescence. Thus, we propose that the spatial distribution of CD81 during the HSC recovery phase drives proliferative HSC to quiescence, and is important to preserve the self-renewal properties. Here, we show that lack of CD81 leads to loss of HSC self-renewal, and the clustering of CD81 on HSC membrane results in deactivation of Akt, which subsequently leads to nuclear translocation of FoxO1a. Thus, CD81 functions as part of a previously undefined mechanism that prohibits excessive proliferation of HSCs exposed to environmental stress.

## Abstract 2

A protein that is thought to orchestrate the distribution of other signaling molecules on the cell membrane, CD81, is critical to maintaining the functional integrity of hematopoietic stem cells during their regeneration.

## Author Summary

Hematopoietic stem cells (HSCs) remain dormant in the bone marrow until needed to replenish the hematopoietic system, at which point they are stimulated to proliferate extensively, undergoing both regeneration (self-renewal) and differentiation. Self-renewal is key to maintaining an adequate HSC reserve, and return to dormancy after such stimulation is critical, yet still poorly understood. In this study, we report that CD81, a transmembrane organizing protein, is a novel regulator involved in HSC self-renewal. Transplanting HSCs into mice that are lethally irradiated to remove their native HSCs stimulates the transplanted HSCs to proliferate to replenish the hematopoietic system, allowing us to examine whether and how HSCs return to quiescence. HSCs lacking CD81 take longer to return to quiescence after such stimulation, resulting in reduced stem cell function. Conversely, forced CD81 membrane clustering, using an antibody, promotes early return of proliferating stem cells to quiescence and nuclear localization of FoxO1a, a key protein that mediates the cell cycle arrest. CD81 clustering also constrains Akt activity, which orchestrates multiple pathways such as cell proliferation and responses to reactive oxygen species. Treatment of *Cd81*-deficient HSCs with an Akt inhibitor, perifosine, which bypasses the requirement for CD81 in this process, rescues the delay defect of *Cd81*-deficient HSCs. Together, our data demonstrate that CD81 is critical to maintaining the functional integrity of HSCs during regeneration, and it is acting through Akt to influence its downstream pathways that govern cell cycle progression.

## Introduction

Hematopoietic stem cells (HSCs), which represent around 1/10^4^ to 1/10^5^ bone marrow cells, can proliferate to replenish the hematopoietic system after its exposure to environmental stresses such as infection, ablative chemotherapy, or irradiation. The stem cell compartment is maintained by self-renewal, in which HSCs generate daughter cells that retain stem cell function after cell divisions. The delicate balance maintained between differentiation and self-renewal necessitates a constellation of intrinsic and extrinsic regulatory mechanisms that are still not well understood. The vast majority of HSCs possess a dormant phenotype (G0 phase of the cell cycle) [1] and slow cycling kinetics (prolonged G1 phase) [2], enforced by regulatory mechanisms that inhibit cell cycle progression from a quiescent stage to active proliferation [3]. In the face of proliferative stimuli, however, these controls are overridden to allow the stem cells to perform their regenerative functions, posing an intriguing question: how do the self-renewing HSCs re-enter a quiescent state? Although extrinsic signaling from cytokines has been proposed to facilitate HSC quiescence [4]–[6], the mechanisms that intrinsically dictate cell cycle exit in HSCs remain largely undefined.

We previously identified several cohorts of genes that are preferentially expressed in HSCs during proliferative and quiescent states [7], and are thus candidates for playing a role in regulation of self-renewal. The gene encoding CD81 (also called Tapa-1), a cell surface transmembrane protein that belongs to the tetraspanin family, emerged from that study as one of the most consistently upregulated molecules in HSCs exposed to proliferative stress [7]. This protein is found in a variety of tissues and has been shown to regulate cell migration, adhesion, and fusion, as well as proliferation and pathogen entry [8]. It is widely expressed in the murine hematopoietic system [9], where its roles include cell-cell interaction, lymphocyte activation, and leukocyte adhesion [10]. This functional versatility and the upregulated expression pattern of *Cd81* in proliferating HSCs led us to hypothesize that this tetraspanin molecule has a specific function in HSC fate determination, particularly during active stem cell regeneration and proliferation.

Serial competitive transplantations provide extreme proliferative stress for HSCs, in which the functional integrity of HSCs has to be delicately maintained and tightly controlled such that an imbalance of cell cycle progression (reviewed in [3]) or uncontrollable level of reactive oxygen species (ROS) [11] compromise the ability of HSCs to regenerate. Akt/Pkb, a serine/threonine kinase, is found to be a key regulator in maintaining HSC integrity. Constitutive activation of Akt in HSCs leads to HSC hyperproliferation and loss of HSC engraftment in primary competitive transplantation (the first round of transplantation) [12], a similar phenotype to that of HSCs lacking Pten, a negative regulator in the Akt pathway [13]. HSCs lacking both *Akt1* and *Akt2* show an engraftment defect in the third competitive transplantation (a third round of transplantation using donor-derived HSCs or bone marrow) [14]. Interestingly, loss of molecules downstream of Akt, such as Atm, FoxOs, and p21, results in similar phenotypes. *Atm* ^−/−^ HSCs failed to engraft long-term in competitive transplantation assays [15]. HSCs lacking FoxO3a show a significantly decreased engraftment in the secondary competitive transplantation [16], while *Foxo1/3/4* ^−/−^ HSCs present a much more severe engraftment defect such that they fail to engraft in the primary transplantation [17]. In addition, HSCs lacking *p21^Cip1/Waf1^*, a cyclin-dependent kinase inhibitor downstream of Akt, exhaust at the 4^th^ transplantation [18].

Here we show, by using *Cd81* ^−*/*−^ HSCs in competitive transplantation assays and monoclonal antibody treatment to induce CD81 clustering on the HSC membrane, that CD81 paces the return of proliferating HSC to a quiescent state. We also demonstrate that CD81 mediates this effect by downregulating the activation state of Akt and subsequently promoting FoxO1a translocation into the nucleus, where it induces cell cycle suppression. Moreover, with the treatment of perifosine, an Akt inhibitor, the engraftment defect of *Cd81* ^−*/*−^ HSCs is corrected, indicating that CD81 is involved in maintaining HSC function integrity through Akt pathways. In addition, we show here the expression of p19, a downstream CDK inhibitor of FoxO proteins [19], is significantly lower during the regeneration of *Cd81* ^−*/*−^ HSCs. In addition, the expression of oxidative responsive genes is significantly decreased in quiescent *Cd81* ^−*/*−^ HSC post-regeneration, suggesting *Cd81* ^−*/*−^ HSCs are more susceptible to reactive oxygen species (ROS), which may contribute to their compromised function.

## Results

### Self-Renewing HSCs Express CD81 during Stress-Induced Proliferation

**Figure 1:**
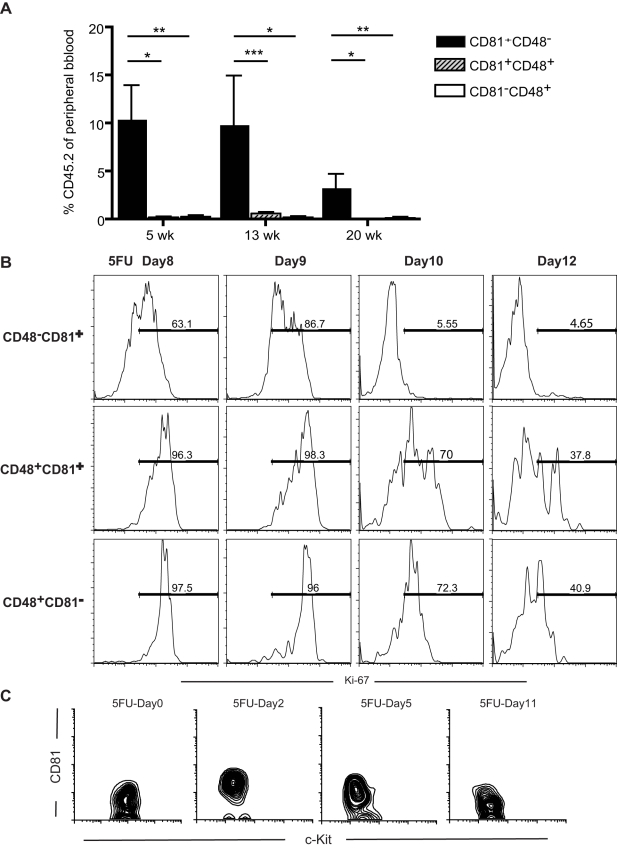
CD81 marks regenerating HSCs that are returning to quiescence. (A) Competitive transplantation assay shows that among the SP cells from the 5FU-treated mice, the CD81^+^CD48^−^ fraction contains most of the stem cell activity, compared with that of the CD81^+^CD48^+^ and CD81^−^CD48^+^ fractions. In this assay, 300Lin^−^SP (Side Population) cells expressing the indicated markers were transplanted with 2.5 × 10^5^ whole bone marrow competitors. The test donor population was derived from CD57Bl/6-CD45.2 mice, while the competitors and recipients were C57BL/6-CD45.1 mice, enabling us to determine the contribution of HSCs to peripheral blood production, using flow cytometry to detect the CD45.2 marker. The values are means ± SD (*n* = 5 per group per time point), **p<0.01, *p<0.05. (B) CD81^+^CD48^−^Lin^−^SP cells show the least expression of Ki-67, a proliferation antigen, at each 5FU time point tested, indicating that CD81^+^CD48^−^ cells are the cells returning to quiescence most rapidly among the Lin^−^SP cells. For each time point, femurs and tibias from 3 to 4 mice were collected in each experiment, and at least two independent experiments were performed for each time point. (C) Expression of CD81 by HSCs (SP cKit^+^Lin^−^Sca1^+^, SP^KLS^) is upregulated when the stem cells are proliferating in response to 5FU stimulation (shown here are 5FU-Day2 and 5FU-Day5). CD81 is expressed at background levels in quiescent HSCs (5FU-Day0 and 5FUDay11), but is upregulated during proliferation (starting at 5FU-Day2).

In our previous study [7], *Cd81* mRNA was sharply upregulated in HSCs (SP^KLS^, c-Kit^+^ Lin^−^ Sca-1^+^ purified from the Side Population of mouse bone marrow cells [20]) after treatment with 5-flurouracil (5FU), a cytotoxic drug that induces HSCs to proliferate. The entire side population (SP) compartment expanded markedly after 5FU induction (Figure S1A), exhibiting a heterogeneous surface expression of CD48, a known marker of HSC differentiation that is absent in unperturbed HSCs [21],[22], and CD81. The presence or absence of CD81 and CD48 defined three subpopulations of the 5FU-stimulated, heterogeneous SP cells (Figure S1B), and the pattern of expression provided an opportunity to further assess the apparent association between CD81 and proliferating HSCs. Interestingly, the CD81^+^CD48^−^ subpopulation was preferentially distributed toward the lower SP (Figure S1B), associated with the most primitive long-term HSC activity [20],[21], consistent with the hypothesis that CD81 plays a functional role in HSC self-renewal. To further test whether CD81 expression was associated with HSC activity after 5FU treatment, we compared the ability of the CD81^+^ or CD81^−^ fractions of SP cells to reconstitute hematopoiesis, using competitive transplantation assays. At 5, 13, and 20 weeks post-transplantation into lethally irradiated mice, the CD81^+^CD48^−^ donor cells showed significantly greater repopulating activity than the CD81^−^CD48^+^ or the CD81^+^CD48^+^ fractions (Figure 1A; the number of CD81^−^CD48^–^ cells is insignificant). Because CD48 expression is associated with differentiation [22], this suggests that these markers delineate a transition between self-renewal and differentiation, with CD81 expression associated with sustained HSC activity. More interestingly, among the three subpopulations, CD81^+^CD48^−^ are the only cells presenting a rapid return to quiescence (Figure 1B), suggesting that HSCs tend to return to quiescence once sufficient progeny are generated.

Further evidence for a role of CD81 in HSC self-renewal came from studies that monitored the expression of CD81 protein by HSCs (SP^KLS^) over the course of 5FU treatment, using an anti-CD81 monoclonal antibody, EAT2. The proliferative response of HSCs to a single dose of 5FU (150 mg/kg) has been established: HSCs begin to proliferate late on day 1 post-treatment and reach maximal proliferation on day 6, returning to quiescence after day 7 [7]. The expression of CD81 correlated closely with the proliferation kinetics of the HSCs (as defined both SP^KLS^ and CD150^+^CD48^−^c-Kit^+^Sca-1^+^Lin^−^). In contrast to its low expression by unstimulated HSCs (5FU-Day 0), the CD81 protein was detected in abundance on days 2, 5, and 8 post-treatment, with a return to background levels by day 11 (Figure 1C).

### Lack of CD81 in HSCs Leads to a Secondary Engraftment Defect

**Figure 2:**
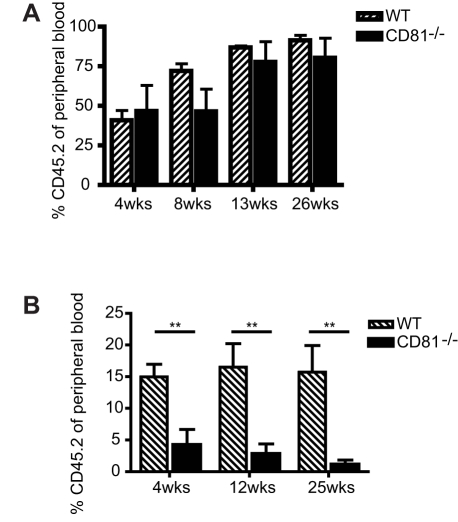
CD81 is essential for maintaining HSC self-renewal. (A) *Cd81* ^−*/*−^ HSCs show comparable engraftment capacity in primary competitive transplantation assays, in which 300 HSCs (CD45.2^+^SP^KLS^) were transplanted with 2.2×10^5^ wild-type (WT) competitors (CD45.1) into lethally irradiated mice (CD45.1^+^). Peripheral blood engraftment was measured at the indicated time points. Mean values ± 50 are shown (*n* = 3). (B) *Cd81* ^−*/*−^ HSCs showed only minimal levels of engraftment in secondary competitive transplantation assays, in which 300 donor-derived HSCs (CD45.2^+^SP^KLS^) were purified from the primary recipients 21 wk after primary transplantation and transplanted along with 2×10^5^ wild-type competitors into lethally irradiated mice. Mean values ± SD are shown (*n* = 10, **p<0.01).

The timing of CD81 expression on proliferating HSCs suggested a role for this molecule in HSC self-renewal, a prediction we sought to test using HSCs purified from CD81 deficient mice (*Cd81* ^−/−^) [23] and wild-type HSCs transplanted into lethally irradiated mice. In the primary competitive transplantation assays (Figure 2A), the engraftment capacity of *Cd81* ^−/−^ HSCs did not differ appreciably from that of the wild-type cells and presented a comparable ability to give rise to blood lineages (Figure S2A). To test whether *Cd81* ^−*/*−^ HSCs were able to generate HSCs in the transplant recipients (self-renewal) we purified HSCs from the primary recipients and examined their function. Despite their ability to differentiate in vitro, as determined by methycellulose assays (Figure S2B), they engrafted secondary transplant recipients only marginally (Figure 1B), indicating a defect in their ability to self-renew. In addition, *Cd81* ^−/−^ and wild-type HSCs showed nearly identical properties in homing assays performed over the first 24 hours of secondary transplantation (Figure S2C), indicating that the hematopoietic repopulation defect shown by the *Cd81* ^−/−^ HSCs is independent of their response to chemokine-guided homing to stem cell niches. It is worth noting that the un-challenged *Cd81* ^−*/*−^ bone marrow not only presents with comparable stem cell composition and cellularity (unpublished data), but the primary transplantation with whole bone marrow cells also exhibited no defect in engraftment (unpublished data), indicating that the defect found in *Cd81* ^−/−^ HSC is specific to the HSC progeny that have gone through self-renewal.

### *Cd81* ^−/−^ HSCs Show Normal Proliferation During Homeostasis But Are Delayed in Returning to Quiescence after Proliferative Stress

**Figure 3:**
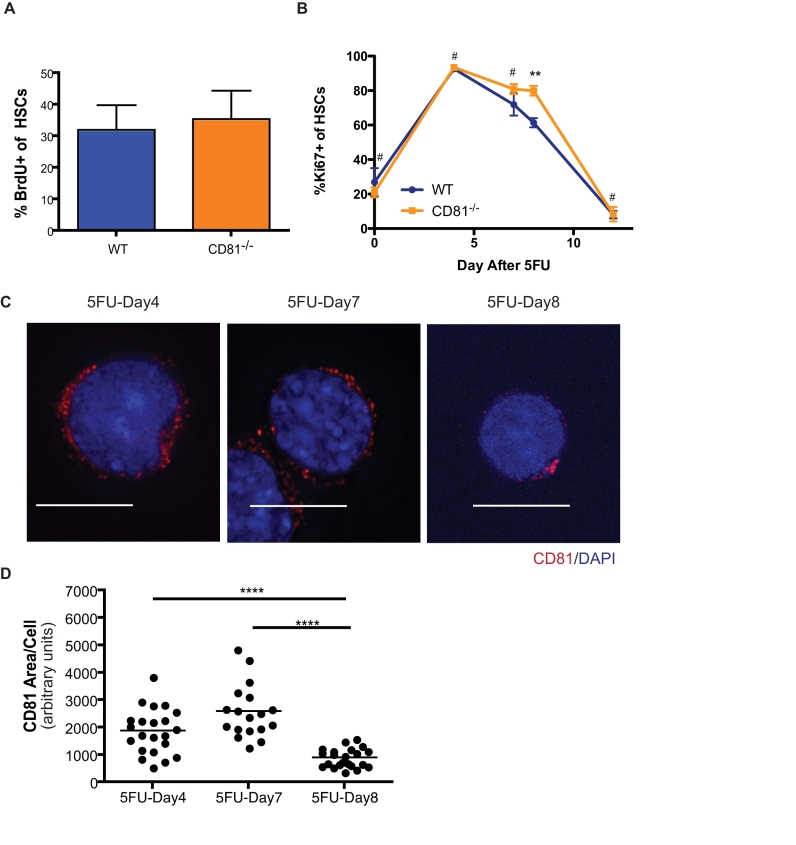
Return to quiescence is delayed in *Cd81* ^−*/*−^ HSCs. (A) Under steady-state conditions, the proportions of proliferating of *Cd81* ^−*/*−^ and wild-type (WT) HSCs are comparable. In this experiment bromodeoxyuridine (BrdU) labeling assays were performed to determine the fraction of cycling cells in primary recipients transplanted with *Cd81* ^−*/*−^ and wild-type mice. Bone marrow from two to three mice was pooled per analysis and studied over 3 d for the incorporation of BrdU. Mean values ± SD are shown. (B) *Cd81* ^−*/*−^ HSCs show delay in early cell cycle exit (5FU-Day8) but a comparable level of quiescence compared to wild type in the later stages (5FU-Day12). Proliferating HSCs were collected and purified at different time points after 5FU administration and stained for Ki-67, a nuclear antigen associated with cell proliferation. Mean values ± SD are shown (*n* = 3 for day 4, day 7, day 8, and day 12, *n* = 2 for day 0; ^#^ *Cd81* ^−/−^ versus WT, day 0, day 4, day 7, and day 12, not significant; **day 8, *p*<0.01). (C) CD81 forms polarized clusters on HSCs transitioning to quiescence on day 8 after 5FU treatment (5FU-Day8). Scale bars represent 10 µM. (D) Compressed distribution of CD81 on day 8 compared with day 4 or day 7 post-treatment with 5FU. Mean values ± SD are shown (****p<0.0001). Horizontal bars denote medium values. Approximately 20 single cell images were measured each time point with ImageJ, which gave an arbitrary value for the area of CD81 signal within a 1 µM z-section around cell nucleus.

To elucidate the mechanism underlying defective engraftment by *Cd81* ^−/−^ HSCs, we considered that a hyperproliferation phenotype leading to depletion of the stem cell pool is often linked to ineffective secondary or serial transplantations [18],[24],[25]. As a molecule highly expressed primarily during the short window of HSC proliferation, we reasoned that CD81 may specifically exert its function only when cells encounter proliferative stress. We therefore challenged primary recipients of transplantation with wild-type or *Cd81* ^−/−^ HSC transplants with one dose of 5FU (150 mg/kg), and evaluated the proliferative status of the donor-derived HSCs. Under steady-state conditions in the absence of proliferative stress, similar fractions of *Cd81* ^−/−^ and wild-type HSCs were proliferating, determined by bromodeoxyuridine (BrdU) labeling assays (Figure 3A). Comparable proportions of *Cd81* ^−/−^ and wild-type HSCs were also proliferating on day 4 after 5FU treatment, suggesting that CD81 does not participate in the early phase of HSC proliferation stimulated by the drug (Figure 3B). Thereafter, the *Cd81* ^−/−^ cells showed only a modest decline in proliferative activation on day 8 after 5FU treatment, while their wild-type equivalents began a relatively rapid return to quiescence (Figure 3B). Interestingly, *Cd81* ^−/−^ HSCs were able to return to a comparable level of quiescence in the later stage of recovery (5FU-Day12), indicating that CD81 functions to facilitate the recovery of HSCs in the face of 5FU stimulation or other types of proliferation stress. Therefore, it is evident that the defective engraftment in the secondary transplantation is not an exhaustion of *Cd81* ^−/−^ HSCs through over-proliferation. Rather, it is a loss of functional integrity with signs of uncoordinated cell cycle progression.

### A Distinct Clustering Pattern of CD81 Is Associated with HSCs Returning to the Quiescent State

**Figure 4:**
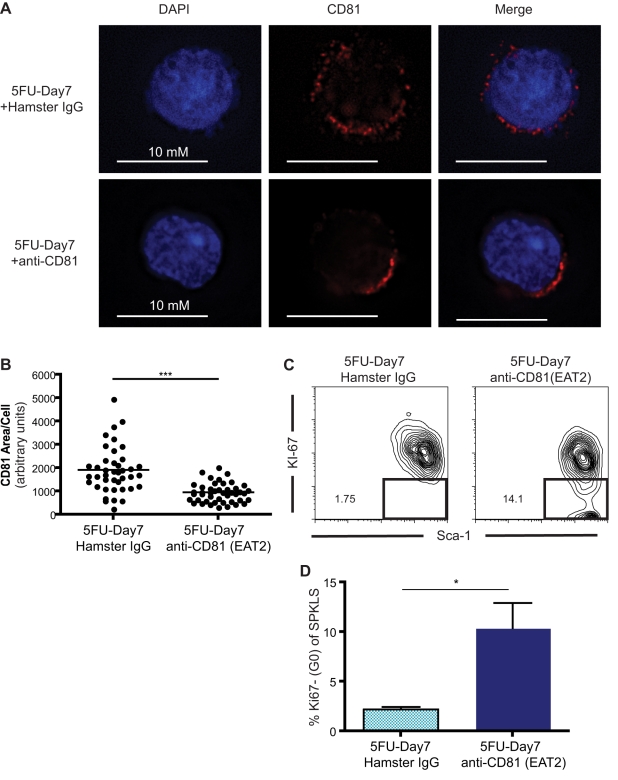
CD81 monoclonal antibody triggers early cell cycle exit. (A) EAT2, a CD81 monoclonal antibody, induces clustering of CD81 on regenerating HSCs (5FU-Day7). With CD81 antibody treatment, the localization of CD81 protein on the wild-type HSC cell membrane shows a distinctively polarized pattern; whereas with the isotype control antibody, the pattern consists of diffuse punctuate dots. (B) The distribution of CD81 on EAT2-treated HSCs is significantly compressed by comparison with the isotype control. In this assay, single cell images were acquired, and the area of CD81 in every acquired image was measured with ImageJ. Horizontal bars denote medium values. Mean values ± SD are shown (***p<0.001). (C) Engagement of CD81 proteins on proliferating HSCs (5FU-Day7) with EAT2 induces a significant fraction of HSC to enter quiescence. Ki-67 staining was used to identify proliferating HSCs. Proportions of quiescent cells are given within the graph. (D) EAT2 induces cell cycle exit. HSCs stimulated with 5FU were treated with EAT2 or hamster IgG (*n* = 3 per group) and assessed for Ki-67 reactivity on day 7 post-treatment. Mean percentages ± SD are shown (*p<0.05).

CD81 is a tetraspanin molecule that is believed to organize membrane domains for signaling molecules and therefore is involved in a variety of signaling pathways downstream of these molecules. We therefore sought to determine if CD81 forms discrete domains in proliferating HSCs. We identified a distinct clustering pattern of the molecule on day 8 after 5FU treatment (Figure 3C,D), which was not observed for other membrane proteins, such as CD29 (unpublished data). CD81 was identified as an anti-proliferation target from monoclonal antibody screenings [26],[27]. It has been reported that engagement of CD81 by a high concentration of monoclonal antibody interferes with cell proliferation [28]. To test whether clustering of CD81 on the cell membranes facilitates quiescence in HSCs, we used an anti-CD81 monoclonal antibody, EAT2 [29], to engage signaling downstream of this tetraspanin molecule. Notably, the antibody-treated proliferating HSCs (5FU-Day7) harbored CD81 protein clustered in patches on the cell membrane, while in the isotype control-treated HSCs, the CD81 proteins were scattered throughout the membrane (Figure 4A). By measuring the area of clustering, we found that the EAT2 treatment promotes tighter clustering of CD81 protein on proliferating (5FU-Day7) HSC (Figure 4B), a day earlier than CD81 naturally coalesces (Figure 3C,D). Finally, binding of the anti-CD81 antibody triggers an early exit of HSCs from the cell cycle, as a significant fraction of HSCs treated with the EAT2 antibody return to quiescence (Figure 4C,D). Thus, cross-linking CD81 molecules with monoclonal antibodies on proliferating HSCs (5FU-Day7) recapitulated what we observed on HSCs in later proliferating stages when returning to quiescence (5FU-Day8), and the clustering pattern of CD81 on HSCs correlated with a quiescence phenotype.

### Manipulating the Distribution of Membrane CD81 Leads to Changes in Akt-FoxO Activity

**Figure 5:**
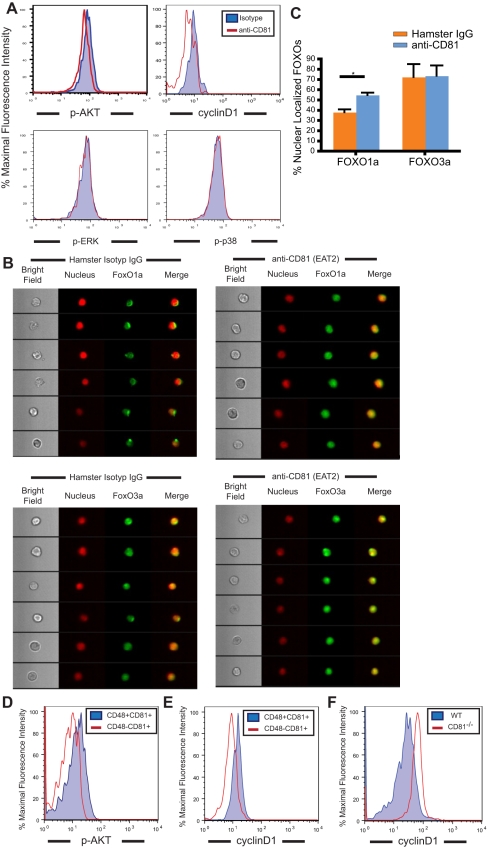
FoxO1a is induced by CD81 clustering, concurrent with deactivation of Akt. (A) The phosphorylation of Akt, an indicator of Akt activation, decreases upon CD81 clustering on proliferating HSCs. EAT2 antibody-treatment of HSCs induces a 20% reduction of phosphorylated-Akt (pAkt), a 30% decrease of CyclinD1 expression, and no decrease of phosphorylated-ERK (p-ERK) or phosphorylated-p38 (p-p38) median fluorescence intensity, compared with results from isotype control-treated HSCs. (B) FoxO1a but not FoxO3a is activated in HSCs in response to EAT2 antibody-mediated CD81 clustering. In EAT2-treated HSCs, FoxO1a shows a nuclear localization phenotype on EAT2-treated cells, whereas in isotype control-treated HSCs the majority of FoxO1a is excluded from nucleus. The localization of FoxO3a, on the other hand, shows no difference in EAT2-treated HSC, compared to the isotype control group. The FoxO translocation was measured by Imagestream. Six representative single cells are shown. (C) Nuclear translocation of FoxO1a in EAT2-treated HSCs is significantly higher in comparison with that in the isotype control group. An established statistic model was employed to calculate degrees of protein nuclear localization with Pearson’s correlation coefficient and generate similarity scores that evaluate the co-localization of FoxO signals with nucleus. Positive nuclear correlation in the single cell images was assigned when the similarity score was more than 1 (more than 100 cells were acquired for each analysis, *n* = 2 per group of treatment; mean values ± SD are shown, *p<0.05). (D) On 5FU-Day8, the level of phospho-Akt in CD81^+^CD48^−^Lin^−^SP cells that are returning to quiescence on 5FU-Day8 is lower than that in the CD81^+^CD48^+^Lin^−^SP cells, a population that is proliferative. Two independent experiments were preformed, and cells from each experiment were collected from 3 to 4 mice. (E) On 5FU-Day8, the CyclinD1 protein expression of the CD81^+^CD48^−^Lin^−^SP cells is lower than that of the CD81^+^CD48^+^Lin^−^SP cells. Three independent experiments were preformed, and cells from each experiment were collected from 3 to 4 mice. (F) Expression of cylinD1 is higher in *Cd81* ^−*/*−^ HSCs in 5FU-Day8 when *Cd81* ^−*/*−^ HSCs show a delay returning to quiescence.

When brought into close proximity, tetraspanin molecules are thought to form microdomains that constrain other resident membrane proteins, ultimately affecting downstream signaling pathways [10]. We therefore investigated the downstream signaling cascade initiated by CD81 clustering in proliferating HSCs, first testing the activation state of ERK (MapK1), p38 (MapK14), JNK (MapK8), and Akt, which were reported to participate in signaling pathways downstream of CD81 after its stimulation by various means [30],[31]. At 1 h after stimulation with EAT2 antibody, only Akt showed a significant reduction (20%) in its phosphorylation state in response to antibody-induced CD81 clustering (judged by the ratio of median fluorescence intensity of phospho-Akt in EAT2-treated HSC to that in isotype-control antibody-treated HSCs) (Figure 5A). More importantly, the expression of CyclinD1, a cell cycle regulator downstream of Akt, was found to decrease by 30% (Figure 5A). This suggests that EAT2-induced CD81 clustering on HSCs leads to a downregulation of Akt activity, which subsequently represses HSC proliferation via CyclinD1 reduction.

The FoxO transcription factors, which are suppressed by Akt during cell proliferation, are essential for HSC self-renewal [32]. Activated Akt phosphorylates FoxO proteins, which are then restricted from entering the nucleus. A return of HSCs to quiescence should thus involve deactivation of Akt and nuclear entrance by FoxOs. To examine whether the Akt deactivation we observed resulted in nuclear localization of any FoxO proteins and suppression of HSC proliferation, we utilized Imagestream flow cytometry [33] to visualize the localization of FoxO1a and FoxO3a and to quantify the number of HSCs with FoxO nuclear localization during FACS analysis. We discovered that FoxO3a was localized in the nucleus regardless of antibody treatment (Figure 5B), while FoxO1a translocated from the cytoplasm to the nucleus after anti-CD81 antibody stimulation (Figure 5B). The nuclear localization of FoxO1a in EAT2-treated HSCs was significantly higher than that in the isotype control-treated HSCs (Figure 5C).

In addition, in the experiments that identify CD81 as a marker for regenerating HSCs that are returning to quiescence (Figure S1A), we observed that the CD81-expressing HSCs (CD81^+^CD48^−^Lin^−^SP cells) correlate with a lower phospho-Akt level (Figure 5D) as well as a lower expression of CyclinD1 protein (Figure 5E). More importantly, *Cd81* ^−*/*−^ HSCs express a higher level of CyclinD1 protein during HSC regeneration (Figure 5F), indicating that HSCs utilize CD81 to manage their recovery from proliferative stimuli by down-regulating Akt activity, and subsequently the protein expression of CyclinD1.

### Perifosine, an Alkylphospholipid, Rescues the Engraftment Defect of *Cd81* ^−*/*−^ HSCs

**Figure 6:**
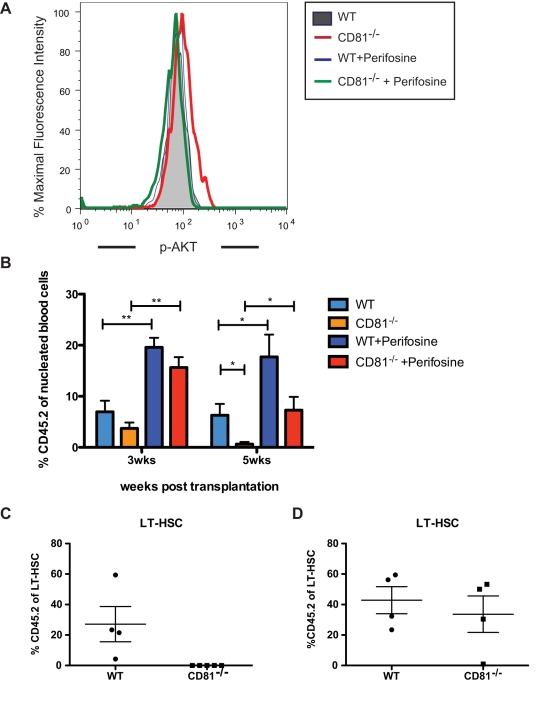
The engraftment defect of *Cd81* ^−/−^ HSCs in the secondary transplantation can be rescued by perifosine. (A) *Cd81* ^−/−^ HSCs present an elevated phospho-Akt, when comparing to the regenerating WT HSCs at the same time point of 5FU-stimulated regeneration; while the level of phospho-Akt can be suppressed with an in vitro perifosine treatment. Two independent experiments were performed. (B) A single perifosine treatment during the recovery phase of the primary transplantation rescues the secondary engraftment defect of *Cd81* ^−/−^ HSCs in the peripheral blood compartment. After stable engraftment, 500 donor HSCs (c-Kit^+^Sca-1^+^Lin^−^CD150^+^CD48^−^CD45.2^+^) from the primary recipients were transplanted into lethally irradiated recipients (CD45.1) with 3×10^5^ WBM competitor cells. The perifosine-treated *Cd81* ^−/−^ HSCs showed viable peripheral blood engraftment at the time when the engraftment of untreated *Cd81* ^−/−^ marrows starts to diminish. Mean values ± SD are shown (*n* = 5 per group of treatment, *p<0.05, **p<0.01). (B) The engraftment defect in the secondary transplantation originates in the HSC compartment. Secondary recipients transplanted with *Cd81* ^−*/*−^ HSCs lack donor-derived HSCs. (C) The secondary engraftment defect of *Cd81* ^−*/*−^ HSCs are rescued by one dose of perifosine. Perifosine was given to the primary recipients at day 7 after the initial transplantation. Ten weeks later, to allow HSCs to regenerate after the primary transplantation, HSCs were purified from the primary recipients and 500 of these donor-derived (*Cd81* ^−*/*−^) HSCs were transplanted along with 3×10^5^ competitor cells into lethally irradiated recipients. Twenty weeks later, the donor HSC-derived progeny in these secondary recipients were examined. Mean percentages ± SD are shown (*p<0.05, *n* = 4 in the wild type control, *n* = 5 in the *Cd81* ^−*/*−^ group, *n* = 4 in the wild type treated with perifosine, *n* = 4 in the *Cd81* ^−*/*−^ treated with perifosine). Figure 5B shows the groups with no perifosine treatment while Figure 5C shows the groups with perifosine treatment. The data from Figure 5B and 5C are from the same cohort but shown separately.

We next investigated the activation level of Akt in the *Cd81* ^−*/*−^ HSCs by measuring the phospho-Akt level and found that the *Cd81* ^−*/*−^ HSCs possess a higher phospho-Akt on 5FU-Day8 when they present a hyper-proliferating phenotype (5FU-Day8). The activity of Akt has been shown to be tightly associated with the proliferation state of HSCs such that constitutive activation of Akt leads to their hyper-proliferation [12] while HSCs lacking both Akt1 and Akt2 were more quiescent [14]. However, the consequences of both the gain and loss of Akt function was to perturb the regeneration power of HSCs such that constitutive activation of Akt resulted in HSC exhaustion [12] and Akt1/Akt2 loss resulted in defective hematopoiesis [14]. We therefore reasoned that Akt activity is tightly controlled during HSC self-renewal, and perturbing Akt activity during the recovery phase of HSC after stress may lead to altered HSC activity. Thus, we incubated recovering HSCs with perifosine, an Akt inhibitor, at the stage when *Cd81* ^−*/*−^ HSCs show higher proliferation and higher expression of CyclinD1. At 30 min after the perifosine treatment (2 µg/ml), we found it returns the phospho-Akt to a comparable level with WT cells (Figure 6A). We administered perifosine to recipients during their recovery phase of transplantation (50 mg/kg, 7 d post-transplantation) when HSCs are presumably recovering and returning to homeostasis. Perifosine is an alkylphospholipid that is thought to incorporate into cell membranes, limit the accessibility of membrane signaling domains for Akt, and subsequently block Akt activation via phosphorylation [34]. Ten weeks later, when the transplant recipients were stably engrafted, the regenerated marrow was extracted and transplanted into secondary recipients. This single administration of perifosine rescues engraftment of donor HSC-derived progeny as functionally measured by their ability to contribute to peripheral blood regeneration in secondary transplant recipients (Figure 6B). Moreover, while the regeneration of *Cd81* ^−*/*−^ HSCs and their progenitors are completely diminished in the untreated group (Figure 6C and Figure S4A), those of perifosine-treated *Cd81* ^−*/*−^ HSCs give rise to comparable number of progeny (Figure 6C and Figure S4B).

Interestingly, the treatment of perifosine that corrects the level of phospho-Akt in *Cd81* ^−*/*−^ HSCs does not bring their proliferation state to a comparable level with WT HSCs (unpublished data). This indicates that although perifosine may have been limiting Akt activity through blocking Akt from the signaling domains on membrane as we propose CD81 may be acting, the inhibitory kinetics are distinct from each other. Nevertheless, the dramatic rescue of the *Cd81* ^−*/*−^ phenotype in the secondary recipients suggests that CD81 functions at least in part via limiting Akt activity during HSC self-renewal.

### The Expression of CDK Inhibitors and Oxidative Stress Genes Are Altered in *Cd81* ^−/−^ HSCs

**Figure 7:**
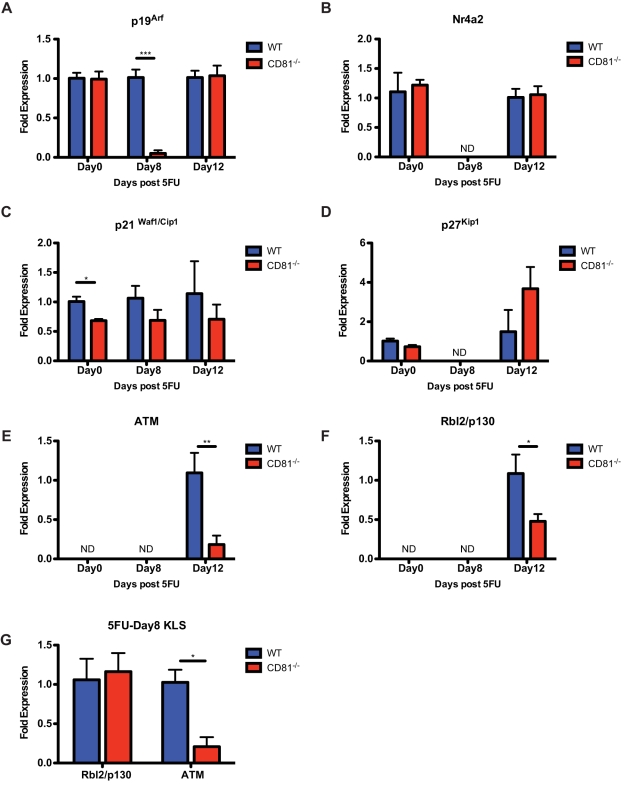
*Cd81* ^−*/*−^ HSCs exhibit low expression of CDK inhibitors and oxidative stress genes during homeostasis and regeneration. (A) Proliferating *Cd81* ^−*/*−^ HSCs (5FU-Day8) express a significantly lower level of *p19^Arf^* while quiescent HSCs (those on 5FU-Day0 and 5FU-Day12) do not. (B) The expression of *Nr4a2* is comparable in quiescent WT and *Cd81* ^−*/*−^ HSCs but not detectable in proliferating HSCs. (C) *Cd81* ^−*/*−^ HSCs in homeostasis present a significantly lower expression of *p21^waf1/cip1^* while those in regeneration do not. (D) The expression of *p27Kip* in both WT and *Cd81* ^−*/*−^ quiescent HSCs are comparable, albeit undetectable in proliferating HSCs. (E and F) The expression of *Atm* and *p130*, the genes that respond to oxidative stresses, are only detectable in HSCs (250 to 300 cell equivalents) in a later stage of 5FU-regeneration (5FU-Day12). The expression of both *Atm* and *p130* in *Cd81* ^−*/*−^ HSCs are significantly lower than those in WT HSCs. (G) On 5FU-Day8, the less primitive hematopoietic progenitors (cKit^+^Lin^−^Sca1^+^, KLS) possess detectable level of *Atm* and *p130* expression, and the expression level of Atm in the *Cd81* ^−*/*−^ progenitor cells is significantly lower than that in the WT cells. Mean percentages ± SD are shown (duplicate or triplicate measurement was undertaken with at least two biological replicates, *p<0.05, **p<0.01, ***p<0.005).

The correlation between the loss of a tightly regulated recovery rate and the engraftment defect of *Cd81* ^−/−^ HSCs after proliferation stimuli suggests that CD81 is essential to maintain the function integrity of regenerating HSCs. We thus sought to determine the expression of CDK (cyclin dependent kinase) inhibitors and oxidative stress genes in HSCs in homeostasis (5FU-Day0) and during regeneration (5FU-Day8 and 5FU-Day12). While we found that the expression of the CDK4/CDK6 inhibitors, *p15*, *p16*, and *p18* [35], was not detectable from 250–300 cell equivalents at all time points (unpublished data), the expression of *p19^Arf^*, a FoxO-dependent CDK inhibitor, is significantly lower in *Cd81* ^−/−^ HSCs at the early recovery phase (5FU-Day8) but comparable in quiescent cells (Figure 7A). In addition, the expression of a CyclinE-CDK2 inhibitor, *p21^Waf/Cip1^* [35], in *Cd81* ^−/−^ HSCs was found lower only during homeostasis (Figure 7C), suggesting an inherently imbalanced cell cycle control. However, the expression of another CyclinE-CDK2 inhibitor, *p27^Kip1^* [35], was found unaltered or undetermined (Figure 7D). Additionally, the expression of *Nr4a2*, a recently identified transcription factor regulating early G1 progression [36], was also comparable in quiescent HSCs (albeit undetectable in regenerating HSCs) (Figure 7B), indicating loss of CD81 only impacts part of the cell cycle regulatory circuitry.

The significantly low level of *p19^Arf^* (Figure 7A) in regenerating *Cd81* ^−/−^ HSCs and the translocation of FoxO1a in response to the CD81 monoclonal antibody modulation (Figure 5B and C) suggest altered responses to oxidative stress in the *Cd81* ^−/−^ HSCs. Akt and FoxO proteins have been found to be essential to maintain HSC identity and function through mediating their resistance to oxidative stress [14],[17],; while the expression of *p19^Arf^* was found to be upregulated in response to oxidative stress and was blocked in hematopoietic progenitors lacking *Atm* (Ataxia Telangiectasia Mutated protein) [11]. Thus, we sought to measure the expression of genes that respond to oxidative stresses, *Atm* and *p130/Rbl2*. The expression of both genes was found to be low in homeostatic HSCs such that they are not detectable in 250 to 300 cell equivalents of RNA. However, at the later HSC regeneration stage (5FU-Day12) when cells have returned to quiescence (Figure 3B), the expression of both genes becomes detectable and their expression level in *Cd81* ^−*/*−^ HSCs was significantly lower (Figure 7E and F).

To further investigate whether oxidative stress may contribute to the defective regeneration phenotypes of *Cd81* ^−/−^ HSCs, we purified donor-derived hematopoietic progenitor cells from the regenerating bone marrow (5FU-Day8) and analyzed the expression level of both *Atm* and *p130/Rbl2*. Atm but not p130/Rbl2 was found to be at a significantly lower level in *Cd81* ^−/−^ hematopoietic progenitors (Figure 7G). Taken together, these findings suggest the oxidative stress may contribute to compromise the function and integrity of *Cd81* ^−*/*−^ HSCs post-hematopoietic regeneration.

## Discussion

It is well accepted that regulation of cell cycle progression in HSCs is critical to maintain stem cell activity [3]; however, there is only scant understanding of the mechanisms by which proliferating HSCs return to quiescence. The results of our study delineate a novel mechanism in which CD81, a stress-responsive membrane protein, is upregulated in HSCs exposed to proliferative stimuli and then acts to inhibit their otherwise unrestrained division. The severe self-renewal defect of *Cd81* ^−*/*−^ HSCs after robust proliferative stress such as transplantation suggests that the role of CD81 in HSC self-renewal is transient and constrained in time.

The key feature of this mechanism seems to be the ability of CD81 molecules to form specialized microdomains on the HSC surface. These domains have been identified on other cell types, such as lymphocytes and astrocytes, where they provide scaffolds for signaling molecules and orchestrate the interactions of membrane-associated proteins with effector molecules to initiate signaling cascade that can either induce or inhibit cell proliferation [37]. Interestingly, we found that CD81 forms a distinct patch-like, polarized pattern when HSCs are returning to quiescence. The membrane distribution of CD81 protein can be manipulated to form clusters with high concentrations of monoclonal antibodies that accelerate the return to quiescence of proliferating HSCs. With this manipulation, only Akt, among several kinases tested including JNK, p38, and ERK, showed a reduction in activation in response to CD81 clustering, suggesting Akt may be closely associated with the CD81 microdomains. Subsequently, we found translocation of FoxO proteins are affected in response to the clustering of CD81. However, with no known ligands for CD81, it is unclear how CD81 is brought together to form patches. As CD81 is thought to be able to interact with itself through its extracellular domain [38], we thus speculate the clustering may be spontaneous when there is an upregulated expression level of CD81 on the cell membrane, such as we observe during HSC regeneration (Figure 1C), which increases the local CD81 density.

Additionally the study with *Cd81* ^−/−^ HSCs in 5FU-stimulated regeneration highlights a time point when HSCs start to return to quiescence after the 5FU proliferative stimuli (5FU-Day8). At this time, *Cd81* ^−*/*−^ HSCs showed a hyper-proliferation phenotype with increased Akt activity, elevated expression of CyclinD1, and decreased RNA expression of *p19^Arf^*, indicating that CD81 acts through Akt pathways during the proliferation phase of HSC regeneration. Moreover, at the stage when HSCs return to quiescence (5FU-Day12), *Cd81* ^−/−^ HSCs show significantly decreased expression of oxidative response genes, *Atm* and *p130/Rbl2*, suggesting that CD81 preserves the functional integrity of regenerating HSCs partly through impacting the oxidative stress response and possibly the intracellular level of ROS (reactive oxygen species). Taken together, these data indicate that CD81 acts through Akt pathways to pace the cell cycle progression, the oxidative stress response, and therefore to preserve the functional integrity of HSCs.

Furthermore, a single in vivo treatment of an alkylphospholipid, perifosine, to the recovering mice (during the primary transplantation) rescues the defective engraftment of *Cd81* ^−*/*−^ HSCs as tested in secondary transplantation. In addition to being an Akt inhibitor, perifosine is found to act through JNK-dependent mechanism and induce caspase-dependent apoptosis [39]. It has been proposed to condition the regiment to treat acute myelogenous leukemia [40],[41]. However, in our study, we have not found an altered activity of JNK in *Cd81* ^−*/*−^ HSCs (unpublished data). We thus suspect that the treatment of perifosine replaces the role of CD81 in the recovering HSCs as microdomains that block signal transduction, and effectively modulates Akt activity. Ultimately, to fully understand how CD81 microdomains communicate with the Akt pathway, it will be necessary to identify their interacting proteins in HSCs in different stages of proliferation. More importantly, our finding that perifosine increases HSCs engraftment may aid to improve the human bone marrow transplantation in treating leukemic patients.

A variety of extrinsic signals provided by the HSC niche have been reported to critically affect the ability of HSCs to maintain stem cell integrity as well as their proliferation status [42]. However, it is unclear how any of these pathways dominantly govern the response of the HSCs to environmental cues. Our study suggests that when HSCs are under proliferative stress, the CD81 microdomains orchestrate the spatial distribution of signaling receptors on membrane as well as membrane-affiliated intracellular molecules on HSCs, and selectively transduce the extrinsic cues from the bone marrow niches. Future studies of the divergent roles of the CD81 microdomains on long-term repopulating HSCs may afford productive targets for the regulation of stem cell growth.

## Materials and Methods

### Mice

All mice were housed at the Baylor College of Medicine according to an AAALAC-approved protocol (Animal Welfare Assurance Number A3823-1). Mice care and treatment were approved by the Institutional Animal Care and Use Committee at the Baylor College of Medicine (IACUC, protocol number AN-2234). 5FU (American Pharmaceutical Partners) was injected intraperitoneally at 150 mg/kg in PBS prior to the assays. In transplantation assays, C57BL/6 mice carrying the CD45.1 allele were used as recipients. *Cd81* ^−*/*−^ mice generated in a 129 background were developed in the Geha lab [23], and were generously provided by R. Kesterson at the University of Alabama, Birmingham, and backcrossed for at least four generations to C57BL/6 mice carrying the CD45.2 allele.

### Flow Cytometry

HSCs used in this study were purified as previously described [43],[44] based on the Hoechst33342 efflux phenotype, which defines a characteristic side population (SP), consisting of hematopoietic stem cells. The hoechst33342-stained whole bone marrow cells were subjected to magnetic enrichment for lineage-negative cells (autoMACS). Surface marker staining for Sca-1, c-Kit, CD81(EAT2), CD48(BCM1), and lineage cocktail (CD4,CD8, B220, Gr-1, Mac-1, and Ter119, BD Bioscience) was performed as previously described [7]. Briefly, 2 ng/ml antibodies were used to stain cells at the concentration of 1×10^8^ cells/ml. Because HSCs are known to upregulate Mac-1 expression during proliferation, the marker was excluded from the lineage cocktail of 5FU-treated bone marrow cells. HSCs were purified from the side population with a Mo-Flo instrument (Beckman Coulter) based on the immunophenotype-SP, c-Kit^+^, Lin^−^, and Sca-1^+^ (SP^KLS^) unless otherwise specified. In the EAT2 antibody-treatment experiment series, HSCs were purified based on the expression of SP, Sca-1^+^, and Lin^-^ (SP^SL^), for the whole bone marrow SP^KLS^ and SP^SL^ populations are highly overlapped. In the homing assays, hematopoietic progenitors are defined as c-Kit^+^Lin^−^Sca-1^+^ (KLS). In the stem cell and progenitor assays, the surface markers used to define each compartment are as follows: LT-HSC (c-Kit^+^Sca-1^+^Lin^−^Flk2^−^CD34^−^), ST-HSC (c-Kit^+^Sca-1^+^Lin^−^Flk2^−^CD34^+^), MPP (c-Kit^+^Sca-1^+^Lin^−^Flk2^+^CD34^+^), CLP (Lin^−^IL7ra^+^c-Kit^+^Sca-1^+^), CMP (Lin^−^IL7ra^−^c-Kit^+^Sca-1^−^CD34^+^CD16/32^−^), MEP (Lin^−^IL7ra^−^c-Kit^+^Sca-1^−^CD34^−^CD16/32^−^), and GMP (Lin^−^IL7ra^−^c-Kit^+^Sca-1^−^CD34^+^CD16/32^+^). FACScan and LSRII (BD Biosciences) were used for the analysis of engraftment and cell proliferation. Engraftment was determined by the proportion of CD45.1 and CD45.2 cells in peripheral blood; the multilineage engraftment was based on the detection of B220 (B cells), CD4 and CD8 (T cells), and Gr-1 and Mac-1 (myeloid lineages) [9].

### Short-Term In Vitro Culture and Monoclonal Antibody Treatment of HSCs

In the monoclonal antibody treatment experiment, HSCs were sorted from wild type mice 7 d post-5FU treatment. Cells were then incubated for 30 min with either 20 µg/ml of CD81 antibody (clone EAT2, BD Biosciences, Cat No. 559518) or of an Armenian Hamster IgG, k isotype control (BD Biosciences, Cat No. 553970). Cells were then fixed in 4% paraformaldehyde for later analysis. In the perifosine treatment experiment, HSCs were sorted and cultured in StemSpan SFEM medium (Stem Cell Technologies, Cat No. 09600) containing 10 ng/ml mouse SCF (Invitrogen, Cat No. PMC2111) for 30 min, with or without the presence of 2 mg/ml perifosine (Selleck Chemicals LLC, Cat No. S1037).

### Competitive Transplantation Assay

In the *Cd81* ^−*/*−^ transplantation assays, given numbers (50–300/experimental group) of donor HSCs (CD45.2) were purified, mixed with 2 to 2.5×10^5^ freshly isolated whole bone marrow cells (CD45.1) as competitors, and transplanted into individual lethally irradiated recipients (CD45.1). In the secondary transplantations, 300 donor-derived (CD45.2) HSCs were transplanted with 2×10^5^ CD45.1 competitors into lethally irradiated recipients (CD45.1). In the perifosine rescue experiments, 50 mg/kg of perifosine was injected into primary recipients (CD45.1) 7 d via intra-peritoneal injection after the first transplantation with 1×10^6^ whole bone marrow cell from either wild-type or *Cd81* ^−*/*−^ mice while Stempro34 (Invitrogen, Cat No. 10639-011) plain media (without nutrient supplement) was injected into the control group. 10 wk after the perifosine injection, 500 donor-derived HSCs (CD45.2^+^c-Kit^+^Sca-1^+^Lin^−^CD150^+^CD48^−^ bone marrow cells) were purified and transplanted into lethally irradiated secondary recipients (CD45.1) with 3×10^5^ whole bone marrow cells as competitors (CD45.1).

### Proliferation Assays of HSCs

To assess HSC proliferation under homeostasis, we performed BrdU labeling as previously described [45]. Three days before the analysis, mice were intraperitoneally injected with BrdU (5 mg/30 g of mouse body weight) and given BrdU-containing drinking water 1 mg/ml) for 3 d. Two or three mice per genotype were pooled for each experiment. SP^KLS^ cells were isolated and mixed with 25,000 carrier cells (B220^+^ or Gr1^+^) for BrdU intracellular staining (BrdU Flow kit, BD Biosciences). In the 5FU-stimulated proliferation experiments, Ki-67 staining was employed to measure cell proliferation because BrdU staining is not compatible with 5FU treatment owing to excessive toxicity. The 5FU-treated HSCs were isolated and mixed with 25,000 carrier cells (B220^+^ cells). Samples were then fixed with Cytoperm/CytoFix solution and permeablized with the Cytoperm solution (BD Bioscience) before the Ki-67 intracellular staining with Ki-67 (BD Biosciences, Cat. No. 612472). The background of each Ki-67 staining experiment varied, so that the positive and negative gates were determined with the isotype control in conjunction with the carrier cells in each sample.

### Immunostaining and Deconvolution Microscopy

Single cell images were acquired by applied precision deconvolution microscopy. In the immunostaining experiments, HSCs were first purified from Mo-Flo (Beckman Coulter) and cytospun (Cytopro, Wescor) onto glass slides. Cells were fixed with 4% paraformaldehyde and subjected to immunostaining of surface markers. CD81 expression was detected with the monoclonal antibody EAT2 (BD Biosciences, Cat. No. 559519). For intracellular staining, HSCs were permeabilized by either ethanol or 0.1X Perm buffer IV (BD Biosciences, Cat No. 560746). HSCs were then labeled with either amine reactive Alexa488 carboxylic acid, succinimidyl ester (Invitrogen, Cat. No. A-20100), or Pacific Blue carboxylic acid, succinimidyl ester (Invitrogen, Cat. No. P-10163). HSCs were mixed with spleenocytes as carrier cells before staining. The intracellular staining of phospho-Akt (p-Akt Thr308, clone C31E5E, Cell Signaling Technology), phospho-ERK (p-ERK Thr202/Tyr204, clone D13.14.4E, Cell Signaling Technology), phospho-p38 (p-p38 Thr180/Tyr182, rabbit polyclonal, Cat. No. 9211, Cell Signaling Technology), phospho-JNK (p-SAPK/JNK Thr183/Tyr185, rabbit polyclonal Cat. No. 9251, Cell Signaling Technology), FoxO1a (rabbit polyclonal, Cat. No. ab39670, Abcam Inc.), and FoxO3a (clone 75D8, Cell Signaling Technology) was quantified with an HRP-conjugated goat anti-rabbit antibody, followed by signaling amplification yielding Alexa647 fluorescence. Intracellular staining was then detected with an LSRII instrument (BD Bioscience). In the experiments that compare the level of p-Akt between wild-type and *Cd81* ^−*/*−^ HSCs, cells were stained with Alexa647-conjugated antibody (pAkt Thr308, clone C31E5E, Cell Signaling Technology). The protein expression of CyclinD1 was detected with a FITC-conjugated CyclinD1 antibody (Clone SP4, Abcam Inc.). Differences in protein expression levels were assessed by comparing ratios of median fluorescence intensities. The localization of FoxOs in single HSCs was determined with ImageStream flow cytometry.

### ImageStream Flow Cytometry

The ImageStream is a multispectral imaging flow cytometer that generates high resolution images of cells at a rate of over 100 cells per second [33]. Spectral compensation and background correction was performed, and images were analyzed with the IDEAS image analysis software. To identify and compare HSCs that had been treated with different agents at the same acquisition, we bar-coded the HSCs with either Alexa488 or Pacific Blue carboxylic acid succinimidyl ester. Approximately 50,000 images per sample were collected to obtain the largest number of the ∼0.5% HSC cells present in each sample.

### Statistics

For comparison of treatment group differences, we used the unpaired two-tailed Student *t* test. All error bars indicate standard errors of the mean (SEM), while *p* values indicated with asterisks were considered significant at the *p* = 0.05 level or lower. To analyze the nuclear localization of FoxO transcription factors, we employed an established statistic model that calculates degrees of protein nuclear localization with Pearson’s correlation coefficient [33]. We assigned a positive nuclear correlation an arbitrary value 1, although the positive correlations may range from 0 to 5.

### RNA Isolation and Q-RT-PCR Analysis

HSC collection at each time point: 3,000 to 5,000 donor-derived HSCs (CD45.2^+^SP^KLS^) were collected on 5FU-Day0, while 10,000 HSCs were collected on 5FU-Day8 and 5FU-Day12. Total RNA was isolated from HSCs using RNeasy Mini Kit (Qiagen, Cat. No. 74104) that provides in-column DNaseI treatment during column purification. First strand synthesis was performed with Superscript VILO cDNA synthesis kit that includes SuperscriptIII reverse transcriptase and random hexamers (Invitrogen, Cat. No. 11754-050). An equivalent of 250 to 330 cells were subjected to each Q-RT-PCR reaction. Q-RT-PCR was performed with pre-validated Taqman probe sets (Applied Biosystems) on a 7300 Real-Time PCR system for 55 cycles. A mouse internal GAPD control was included in every reaction for normalization. The threshold cycle was determined with software provided by the manufacturer, and expression was measured for each assay relative to the GAPD internal standard (ΔCt). Assays were performed in triplicate (technical replicates) and each experiment was performed in at least two biological replicates. Relative expression between two cell populations was calculated by subtracting the ΔCt values (ΔΔCt). Fold differences were calculated as 2̂(ΔΔCt) when ΔΔCt > 0 or −(2̂(−ΔΔCt)) when ΔΔCt < 0.

## Supporting Information

Figure S1 CD81 marks regenerating HSCs. (A) The bone marrow side population (SP) expands in response to single administration of 5FU. Under homeostasis, 0.1% of Lineage-depleted bone marrow cells (Lin^−^: CD4^−^, CD8^−^, B220^−^, Mac1^−^, Gr1^−^, and Ter119^−^) are side population (SP), while on day 7 after the 5FU treatment, this percentage increases to 1.8%. (B) CD81^+^SP cells possess a higher Hoechst dye efflux capacity (and hence lower fluorescence), a phenotype associated with stem cell activity, in contrast to CD48^+^SP cells. CD81 and CD48 define discrete subpopulations within the 5FU-Day7 SP population. CD48^+^CD81^−^ marks the lower to tip SP fraction (55% of tip-SP cells), while CD81^−^CD48^+^ marks the upper-to-shoulder fraction (19% of tip-SP cells). Cd81^+^CD48^+^ marks the intermediate SP population (23% of tip-SP cells).
(PDF)

Figure S2 *Cd81* ^−*/*−^ HSCs showed comparable contribution to blood lineages, colony forming ability and homing ability after the primary competitive transplantation. (A) *Cd81^−/−^* HSCs were able to generate comparable portion of blood lineages in the recipients of the primary competitive transplantation, indicating the multilineage differentiation ability of *Cd81^−/−^* HSC is intact. Representative cohort shown here is the blood engraftment at the 14th week post whole bone marrow transplantation (n = 28 for wild-type, n = 29 for *Cd81^−/−^*). (B) Post the primary transplantation, *Cd81^−/−^* HSCs showed comparably ability to generate colonies albeit presenting a defective phenotype in the recipients of the secondary transplantation. After the primary transplantation, single HSCs were purified and placed in M3434 metholcult media for assaying colony-forming ability. 48-96 single cells were scored for colony forming in each set of single M3434 culture. Mean value ± SD findings are shown (n = 3 for both WT and *Cd81^−/−^*). (C) To test whether *Cd81^−/−^* HSCs are defective in their ability to reach the bone marrow niche after the primary transplantation, we performed a homing assay, using *Cd81^−/−^* HSC from the recipient mice of the primary competitive transplantation assay. The CFSE based homing assay was modified from a previous protocol [1]. Briefly, the CD45.2 nucleated bone marrow cells from the original transplant recipients were magnetically purified and labeled with a fluorescent lipid dye, PKH2 Green Fluorescent Cell Linker (Sigma). 2×10^7^ cells were then transplanted lethally irradiated mice. After 12 h, the recipient mice were sacrificed and one leg (a tibia and a femur) was collected for analysis. The homed donor progenitor cells were measured as the percentage of fluorescence-labeled KLS (c-Kit^+^ Lineage- and Sca-1^+^) cells in recipient bone marrow. Mean ± SD findings are shown (n = 4 for wild-type and 3 for *Cd81^−/−^* transplantation). CFSE is the abbreviation of carboxyfluorescein succinimidyl ester, a fluorescence dye labeling cell membrane).
(PDF

Figure S3 Gating schemes of the Ki-67 detection and of the expression level of p-Akt. (A) In the 5FU time course study, proliferating cells of HSCs were measured by the expression of Ki-67. HSCs were purified based on the properties of Hoechst33342 efflux (SP) and marker expression including c-Kit^+^, Sca-1^+^, and Lin- (SPKLS), as well as the expression of CD45 marker that distinguish donor-derived HSCs (CD45.2^+^) from the competitors (CD45.1^+^). Ki-67 gates were drawn based on the internal controls of each analysis, which is non-stimulated spleenocytes. (B) The scheme of color-coated HSCs in the analysis of p-Akt level. Wild-type or *Cd81^−/−^* HSCs were sorted based on the markers: SPKLS and CD45.2^+^CD45.1*^−^*. Cells were then fixed with 4% paraformaldehyde and permeablized with 0.1× Perm Buffer IV (BD Biosciences, Cat No. 560746) during which *Cd81^−/−^* cells were color coated with an amine-reactive pacific blue succinimidyl ester (Invitrogen, Cat No. P10163). (B) Wild-type and *Cd81^−/−^* cells were pooled before the staining and analysis of level of phospho-Akt (p-Akt). The level of p-Akt was detected with either an Alexa647-conjugated phospho-Akt (Thr308) monoclonal antibody (Cell signaling Technology, Cat No. 3375), or non-conjugated phosphor-Akt (Thr308) monoclonal antibody (Cell Signaling Technology, Cat No. 2965) with a HRP-conjugated, goat ant-rabbit secondary antibody that is detected with a Alexa647-tyramide signal amplification kit (Invitrogen, Cat No. T20926). Representative data shown in Figure 6 is the one detected with the Alexa647-conjufated p-Akt antibody.
(PDF)

Figure S4 The engraftment defect of *Cd81* ^−*/*−^ progenitors in the secondary transplantation can be rescued by perifosine. (A) The engraftment defect in the secondary transplantation is found in the progenitor compartments. Analysis of progenitor compartments was preformed as previously described [2],[3]. Secondary recipients transplanted with *Cd81* ^−*/*−^ HSC lack progenitors such as MPP (multipotent progenitors), CMP (common myeloid progenitors, Lin^−^cKit^+^Sca1^−^Il7rα^−^CD34^+^CD16/32^−^), CLP (common lymphoid progenitors, Lin^−^cKit^+^Sca1^+^Il7rα^+^), MEP (megakaryocyte-erythrocyte progenitors, Lin^−^cKit^+^Sca1^−^Il7rα^−^CD34^−^CD16/32^−^), and GMP (Granulocutemacrophage progenitors, Lin^−^cKit^+^Sca1^−^Il7rα^−^CD34^+^CD16/32^+^). (B) The secondary engraftment defect of *Cd81-/-* progenitors is rescued by one dose of perifosine (50 mg/kg). *Cd81* ^−*/*−^ HSCs that had been exposed to the perifosine treatment gave rise to comparable numbers of stem and progenitor progeny, except for a significantly lower CLP engraftment. Mean percentage ± SD are shown (*p<0.05, n = 4 in the wild-type control, n = 5 in the *Cd81* ^−*/*−^ group, n = 4 in the wild-type treated with perifosine, n = 4 in the *Cd81* ^−*/*−^ treated with perifosine). (C) HSC and MPP gating schemes. Bone marrows from secondary recipients that were transplanted with either wild-type or *Cd81* ^−*/*−^ HSCs, with or without perifosine treatment, were analyzed for the donor-derived (CD45.2^+^) HSCs and progenitors. Examples shown here are the gating scheme of LT-HSC (Lin^−^cKit^+^Sca1^+^Flk2^−^CD34^−^), ST-HSC (Lin^−^cKit^+^Sca1^+^Flk2-CD34^+^), and MPP (Lin^−^cKit^+^Sca1^+^Flk2^+^CD34^+^).
(PDF)
