# Supplementary figures and images for: CD81 Is Essential for the Re-entry of Hematopoietic Stem Cells to Quiescence following Stress-Induced Proliferation Via Deactivation of the Akt Pathway
Source: PLoS Biol. 2011 Sep 13;9(9):e1001148. doi: 10.1371/journal.pbio.1001148 (PMC3172193; doi:10.1371/journal.pbio.1001148)

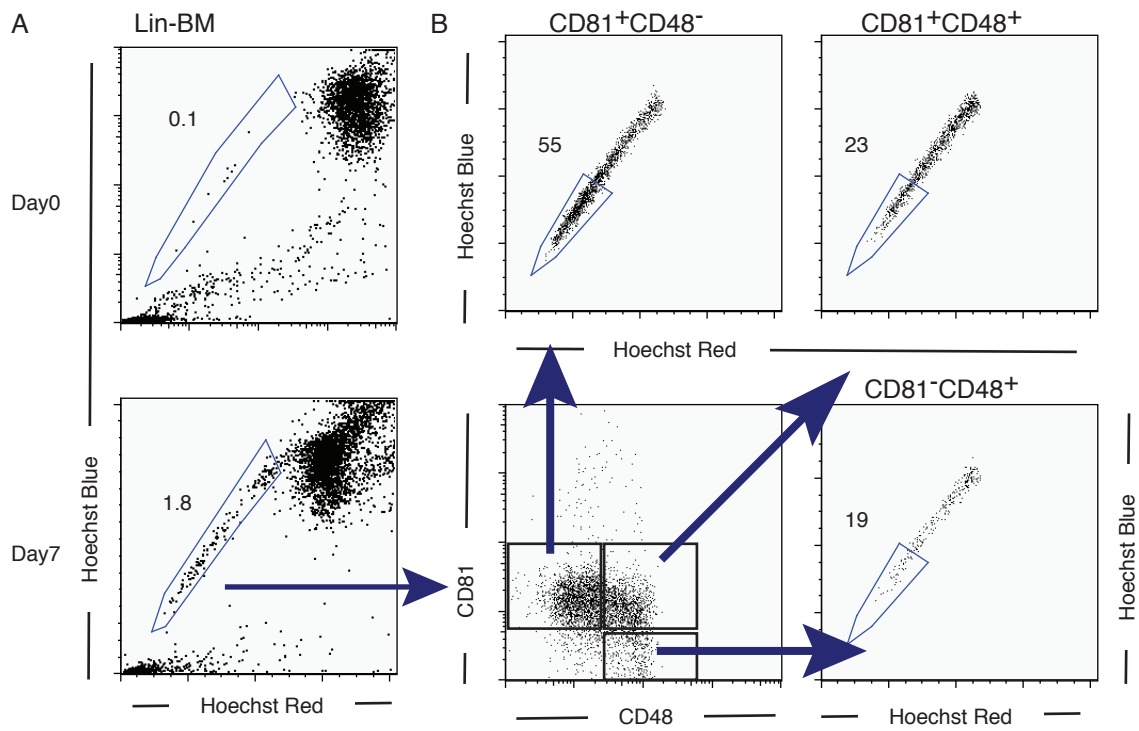

Supplement: Figure S1 — CD81 marks regenerating HSCs. (A) The bone marrow side population (SP) expands in response to single administration of 5FU. Under homeostasis, 0.1% of Lineage-depleted bone marrow cells (Lin−: CD4−, CD8−, B220−, Mac1−, Gr1−, and Ter119−) are side population (SP), while on day 7 after the 5FU treatment, this percentage increases to 1.8%. (B) CD81+SP cells possess a higher Hoechst dye efflux capacity (and hence lower fluorescence), a phenotype associated with stem cell activity, in contrast to CD48+SP cells. CD81 and CD48 define discrete subpopulations within the 5FU-Day7 SP population. CD48+CD81− marks the lower to tip SP fraction (55% of tip-SP cells), while CD81−CD48+ marks the upper-to-shoulder fraction (19% of tip-SP cells). Cd81+CD48+ marks the intermediate SP population (23% of tip-SP cells). (PDF) [file pbio.1001148.s001.pdf]

A

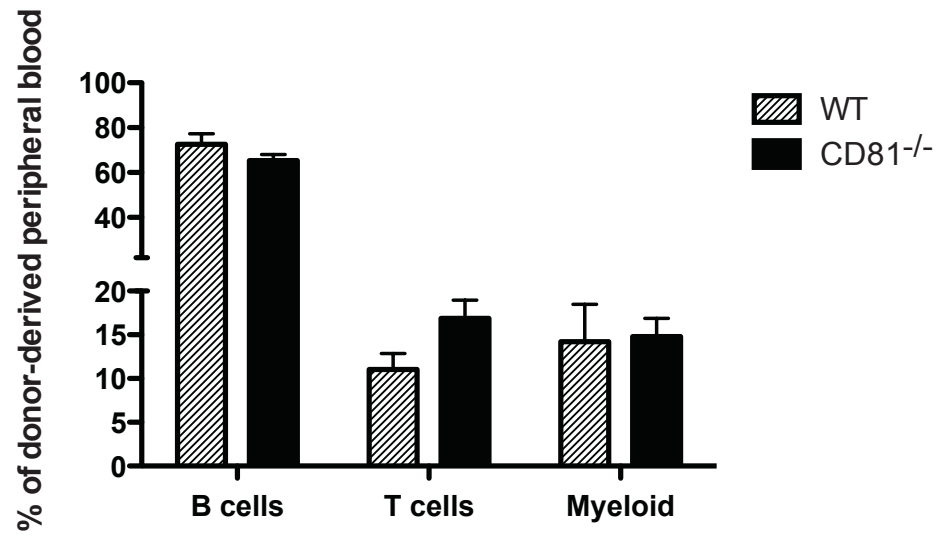

B

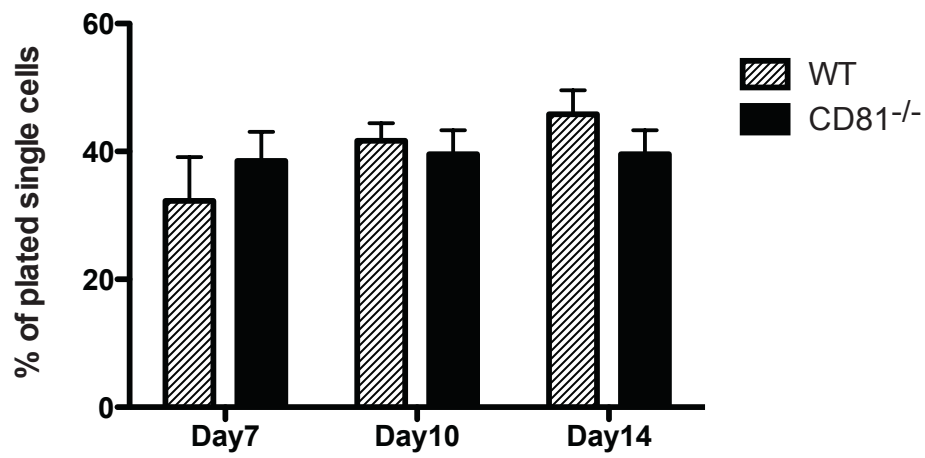

C

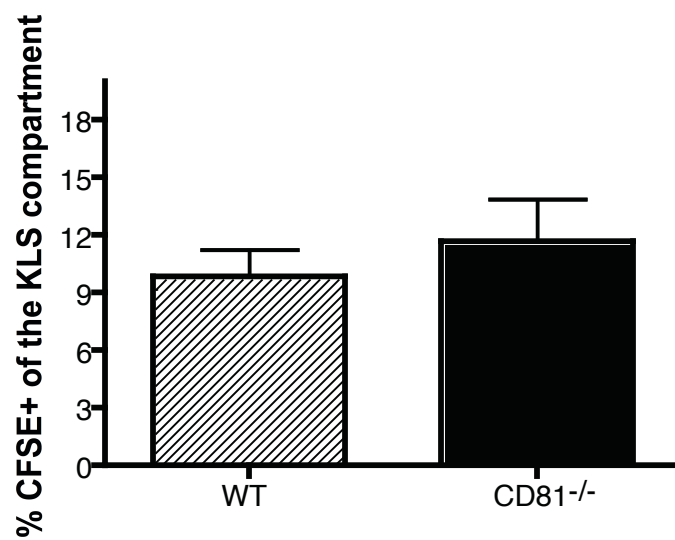

Supplement: Figure S2 — Cd81 −/− HSCs showed comparable contribution to blood lineages, colony forming ability and homing ability after the primary competitive transplantation. (A) Cd81−/− HSCs were able to generate comparable portion of blood lineages in the recipients of the primary competitive transplantation, indicating the multilineage differentiation ability of Cd81−/− HSC is intact. Representative cohort shown here is the blood engraftment at the 14th week post whole bone marrow transplantation (n = 28 for wild-type, n = 29 for Cd81−/−). (B) Post the primary transplantation, Cd81−/− HSCs showed comparably ability to generate colonies albeit presenting a defective phenotype in the recipients of the secondary transplantation. After the primary transplantation, single HSCs were purified and placed in M3434 metholcult media for assaying colony-forming ability. 48-96 single cells were scored for colony forming in each set of single M3434 culture. Mean value ± SD findings are shown (n = 3 for both WT and Cd81−/−). (C) To test whether Cd81−/− HSCs are defective in their ability to reach the bone marrow niche after the primary transplantation, we performed a homing assay, using Cd81−/− HSC from the recipient mice of the primary competitive transplantation assay. The CFSE based homing assay was modified from a previous protocol [1]. Briefly, the CD45.2 nucleated bone marrow cells from the original transplant recipients were magnetically purified and labeled with a fluorescent lipid dye, PKH2 Green Fluorescent Cell Linker (Sigma). 2×10^7^ cells were then transplanted lethally irradiated mice. After 12 h, the recipient mice were sacrificed and one leg (a tibia and a femur) was collected for analysis. The homed donor progenitor cells were measured as the percentage of fluorescence-labeled KLS (c-Kit+ Lineage- and Sca-1+) cells in recipient bone marrow. Mean ± SD findings are shown (n = 4 for wild-type and 3 for Cd81−/− transplantation). CFSE is the abbreviation of carboxyfluorescein succinimidyl [file pbio.1001148.s002.pdf]

A

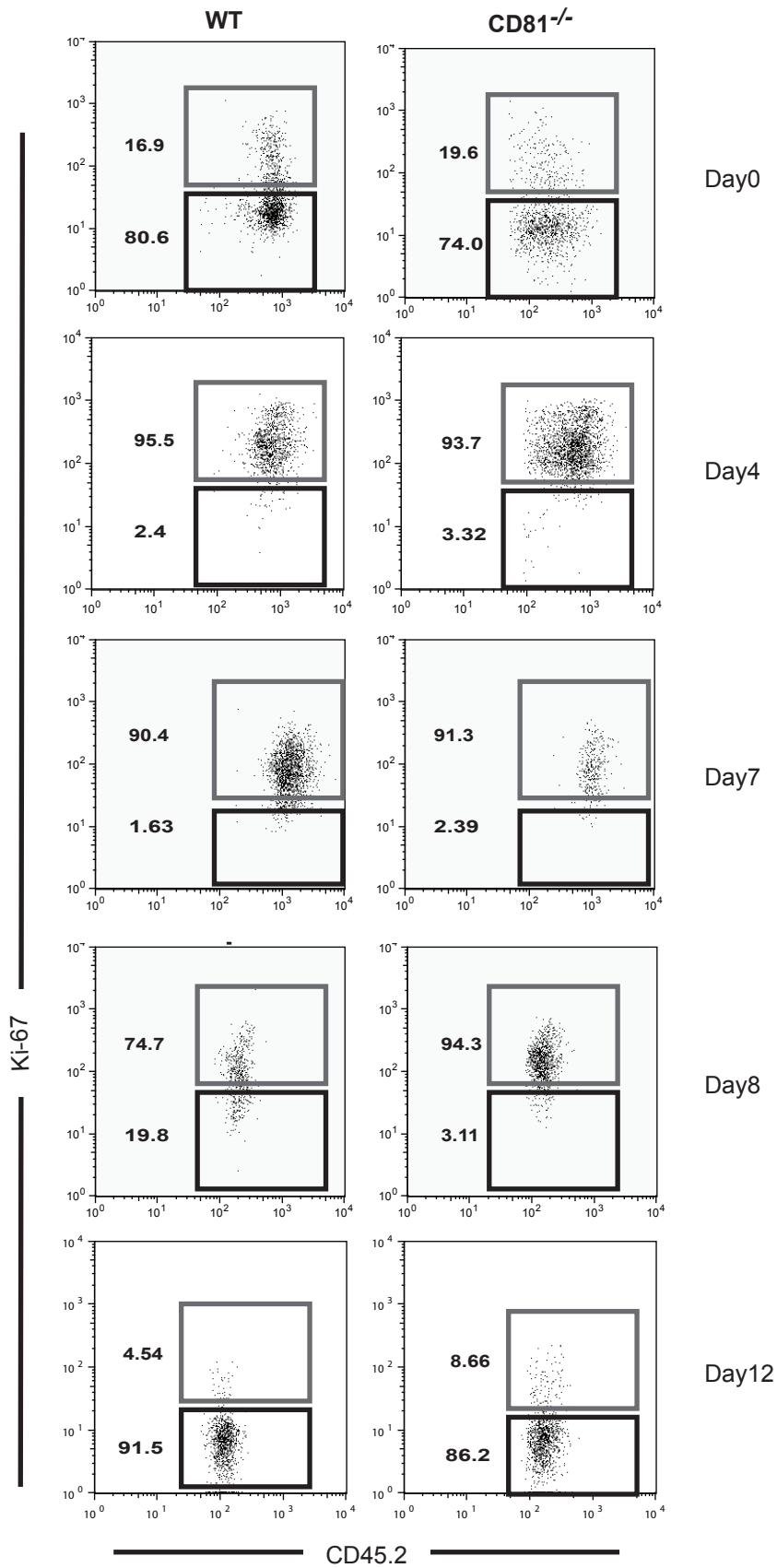

B

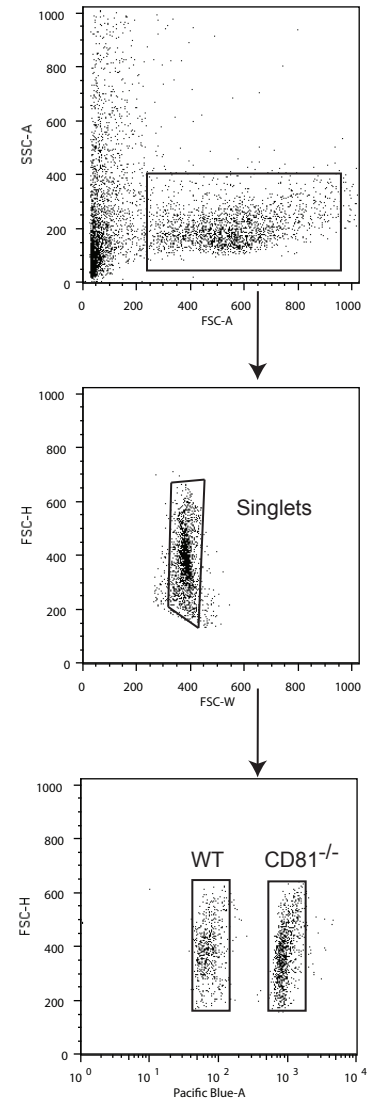

Supplement: Figure S3 — Gating schemes of the Ki-67 detection and of the expression level of p-Akt. (A) In the 5FU time course study, proliferating cells of HSCs were measured by the expression of Ki-67. HSCs were purified based on the properties of Hoechst33342 efflux (SP) and marker expression including c-Kit+, Sca-1+, and Lin- (SPKLS), as well as the expression of CD45 marker that distinguish donor-derived HSCs (CD45.2+) from the competitors (CD45.1+). Ki-67 gates were drawn based on the internal controls of each analysis, which is non-stimulated spleenocytes. (B) The scheme of color-coated HSCs in the analysis of p-Akt level. Wild-type or Cd81−/− HSCs were sorted based on the markers: SPKLS and CD45.2+CD45.1−. Cells were then fixed with 4% paraformaldehyde and permeablized with 0.1× Perm Buffer IV (BD Biosciences, Cat No. 560746) during which Cd81−/− cells were color coated with an amine-reactive pacific blue succinimidyl ester (Invitrogen, Cat No. P10163). (B) Wild-type and Cd81−/− cells were pooled before the staining and analysis of level of phospho-Akt (p-Akt). The level of p-Akt was detected with either an Alexa647-conjugated phospho-Akt (Thr308) monoclonal antibody (Cell signaling Technology, Cat No. 3375), or non-conjugated phosphor-Akt (Thr308) monoclonal antibody (Cell Signaling Technology, Cat No. 2965) with a HRP-conjugated, goat ant-rabbit secondary antibody that is detected with a Alexa647-tyramide signal amplification kit (Invitrogen, Cat No. T20926). Representative data shown in Figure 6 is the one detected with the Alexa647-conjufated p-Akt antibody. (PDF) [file pbio.1001148.s003.pdf]

A

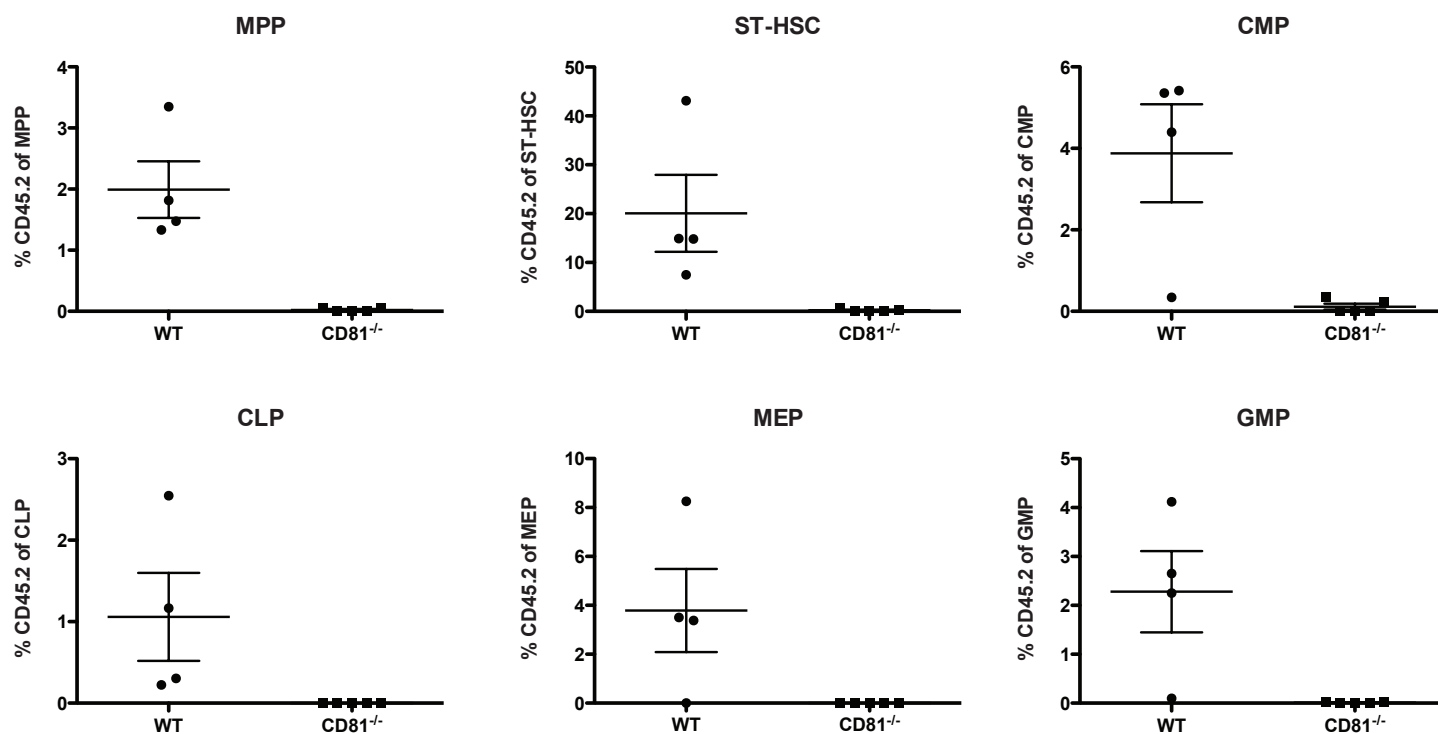

B

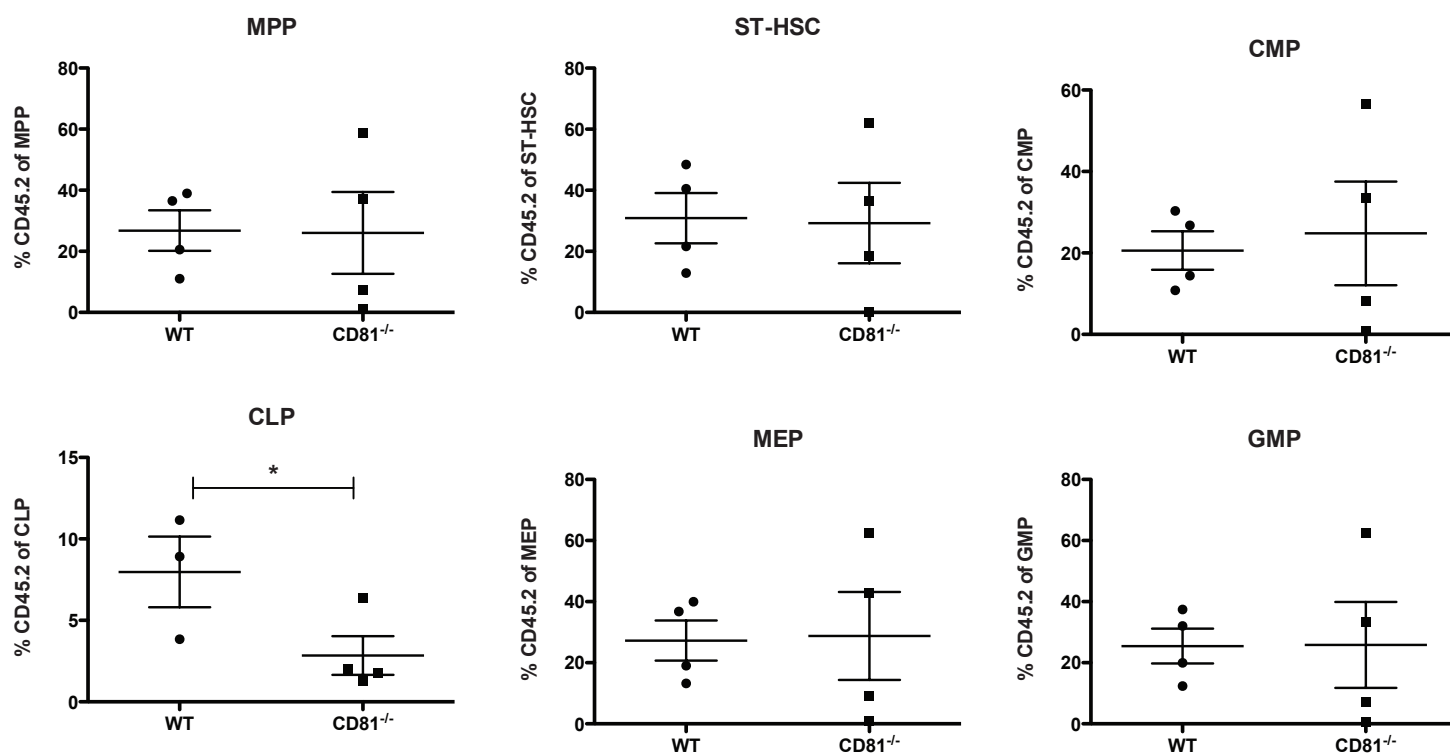

C

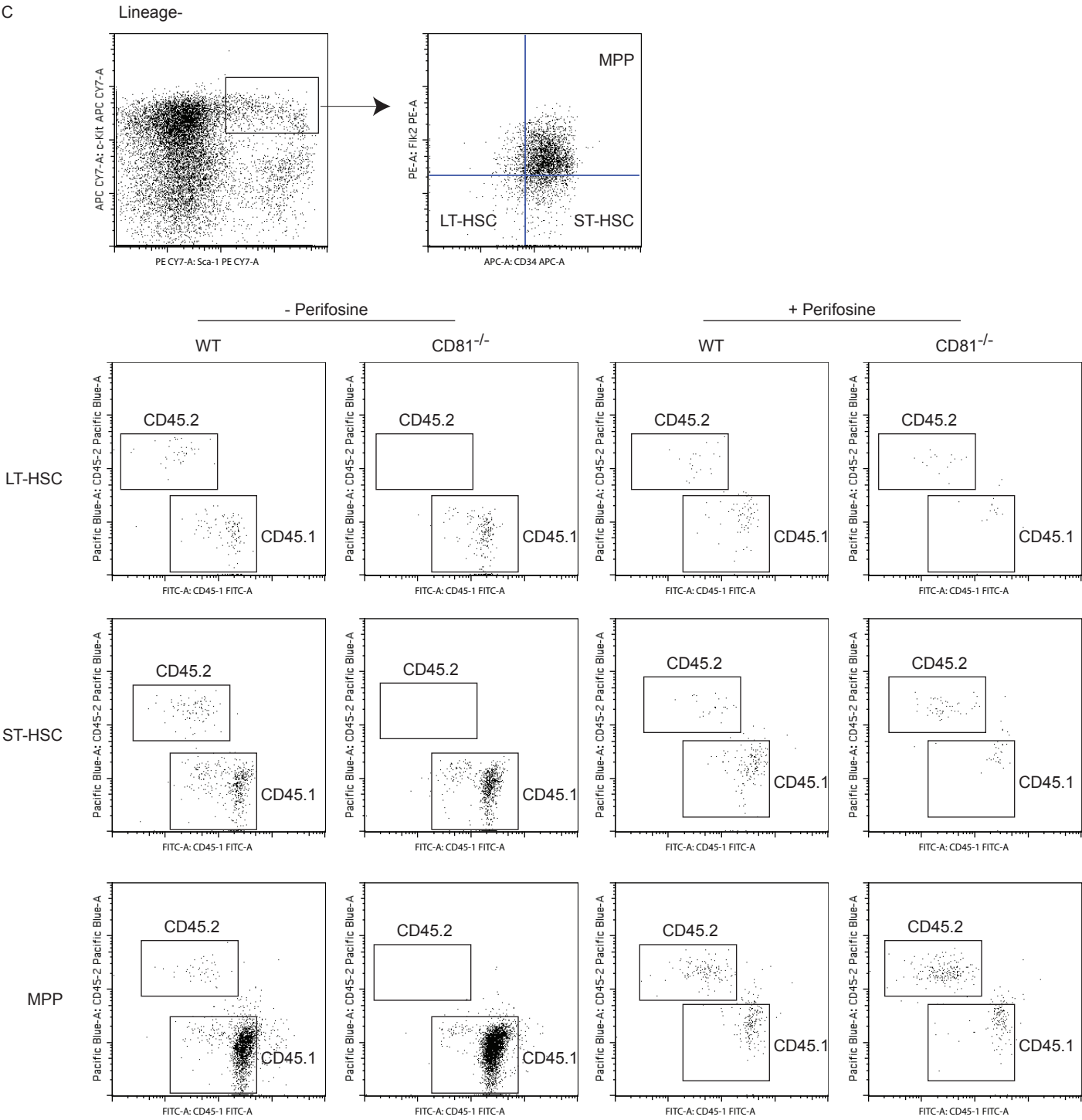

Supplement: Figure S4 — The engraftment defect of Cd81 −/− progenitors in the secondary transplantation can be rescued by perifosine. (A) The engraftment defect in the secondary transplantation is found in the progenitor compartments. Analysis of progenitor compartments was preformed as previously described [2],[3]. Secondary recipients transplanted with Cd81 −/− HSC lack progenitors such as MPP (multipotent progenitors), CMP (common myeloid progenitors, Lin−cKit+Sca1−Il7rα−CD34+CD16/32−), CLP (common lymphoid progenitors, Lin−cKit+Sca1+Il7rα+), MEP (megakaryocyte-erythrocyte progenitors, Lin−cKit+Sca1−Il7rα−CD34−CD16/32−), and GMP (Granulocutemacrophage progenitors, Lin−cKit+Sca1−Il7rα−CD34+CD16/32+). (B) The secondary engraftment defect of Cd81-/- progenitors is rescued by one dose of perifosine (50 mg/kg). Cd81 −/− HSCs that had been exposed to the perifosine treatment gave rise to comparable numbers of stem and progenitor progeny, except for a significantly lower CLP engraftment. Mean percentage ± SD are shown (*p<0.05, n = 4 in the wild-type control, n = 5 in the Cd81 −/− group, n = 4 in the wild-type treated with perifosine, n = 4 in the Cd81 −/− treated with perifosine). (C) HSC and MPP gating schemes. Bone marrows from secondary recipients that were transplanted with either wild-type or Cd81 −/− HSCs, with or without perifosine treatment, were analyzed for the donor-derived (CD45.2+) HSCs and progenitors. Examples shown here are the gating scheme of LT-HSC (Lin−cKit+Sca1+Flk2−CD34−), ST-HSC (Lin−cKit+Sca1+Flk2-CD34+), and MPP (Lin−cKit+Sca1+Flk2+CD34+). (PDF) [file pbio.1001148.s004.pdf]
